# Metabolomics reveals dose effects of low-dose chronic exposure to uranium in rats: identification of candidate biomarkers in urine samples

**DOI:** 10.1007/s11306-016-1092-8

**Published:** 2016-09-15

**Authors:** Stéphane Grison, Gaëlle Favé, Matthieu Maillot, Line Manens, Olivia Delissen, Éric Blanchardon, Isabelle Dublineau, Jocelyne Aigueperse, Sandra Bohand, Jean-Charles Martin, Maâmar Souidi

**Affiliations:** 1 Institut de Radioprotection et de Sûreté Nucléaire (IRSN), PRP-HOM, SRBE, LRTOX, 92260 Fontenay-aux-roses, France; 2 Aix Marseille Université (AMU), NORT, 13005 Marseille, France; 3 Inserm, UMR_S 1062, 13005 Marseille, France; 4 Inra, UMR_INRA 1260, 13005 Marseille, France; 5 MS-Nutrition, Marseille, France; 6 Institut de Radioprotection et de Sûreté Nucléaire (IRSN), PRP-HOM, SDI, LEDI, 92260 Fontenay-aux-roses, France; 7 Institut de Radioprotection et de Sûreté Nucléaire (IRSN), PRP-HOM, 92260 Fontenay-aux-roses, France; 8 AREVA Mines, 92400 Courbevoie, France

**Keywords:** Metabolomics, Chronic, Low dose, Contamination, Uranium, N1-methylnicotinamide

## Abstract

**Introduction:**

Data are sparse about the potential health risks of chronic low-dose contamination of humans by uranium (natural or anthropogenic) in drinking water. Previous studies report some molecular imbalances but no clinical signs due to uranium intake.

**Objectives:**

In a proof-of-principle study, we reported that metabolomics is an appropriate method for addressing this chronic low-dose exposure in a rat model (uranium dose: 40 mg L^−1^; duration: 9 months, n = 10). In the present study, our aim was to investigate the dose–effect pattern and identify additional potential biomarkers in urine samples.

**Methods:**

Compared to our previous protocol, we doubled the number of rats per group (n = 20), added additional sampling time points (3 and 6 months) and included several lower doses of natural uranium (doses used: 40, 1.5, 0.15 and 0.015 mg L^−1^). LC–MS metabolomics was performed on urine samples and statistical analyses were made with SIMCA-P+ and R packages.

**Results:**

The data confirmed our previous results and showed that discrimination was both dose and time related. Uranium exposure was revealed in rats contaminated for 9 months at a dose as low as 0.15 mg L^−1^. Eleven features, including the confidently identified N1-methylnicotinamide, N1-methyl-2-pyridone-5-carboxamide and 4-hydroxyphenylacetylglycine, discriminated control from contaminated rats with a specificity and a sensitivity ranging from 83 to 96 %, when combined into a composite score.

**Conclusion:**

These findings show promise for the elucidation of underlying radiotoxicologic mechanisms and the design of a diagnostic test to assess exposure in urine, in a dose range experimentally estimated to be above a threshold between 0.015 and 0.15 mg L^−1^.

**Electronic supplementary material:**

The online version of this article (doi:10.1007/s11306-016-1092-8) contains supplementary material, which is available to authorized users.

## Introduction

Characterizing the long-term biological effects associated with exposure to chemical pollutants, such as drugs, pesticides and radionuclides, proves to be rather complex when it comes to chronic low-dose contamination. These effects generally include several molecular events involving several metabolic pathways that induce low amplitude effects barely distinguishable from physiological variability. Primary data obtained from cohorts weakly exposed to chemical compounds regularly used in the chemical industry and agriculture recently revealed biological effects without any apparent phenotypic change or morbidity (Bonvallot et al. 2013; Dudka et al. 2014). Unfortunately, in this context the use of conventional clinical analysis combined with univariate statistical analyses is unsuitable for identifying low-dose biological effects, both because the body is an integrated system involving multiple and interconnected forms of complex metabolic regulation and because no phenotypic effect can be observed within narrow time-windows, especially in the low-dose ranges.

Among the various pollutants that have sparked societal concern about the risks of low doses, radionuclides are found dispersed in the environment, both naturally, since they are present in the Earth’s crust, or from anthropogenic sources, such as nuclear fuel cycles or military use (MacKenzie 2000). The dispersion rate depends on their origin (natural, accidental or post-accidental) and geographical location (Hu et al. 2010). Routes of human exposure and contamination include skin injury, inhalation and, often, the ingestion of low concentrations of radionuclides through the food chain and spring water consumption. Radionuclide toxicity is relatively well described for the high-contamination dose ranges, which are associated with characteristic pathological effects (Kathren and Burklin 2008; Papadopoulou and Efthimiou 2009), but remains a controversial topic for low dose ranges, because of the scarcity of investigations (Morgan and Bair 2013). Nonetheless, the health consequences of chronic low-dose exposure to radionuclides are an important public issue because of their potential major societal and political impact. In particular, uranium is naturally present in the earth’s crust and can be found in drinking water at various concentrations, depending on the geological nature of the soil (UNSCEAR 2008). Additionally, a major concern about uranium levels in drinking water is the biological impact of chronic ingestion during sensitive periods such as pregnancy, childhood and old age.

Some in vivo experimental studies in rodents chronically exposed with uranium report molecular imbalances associated, for example, with the cholinergic pathway and oxidative stress in the brain, with the cholesterol metabolism and the liver xenobiotic metabolism, with inflammatory mediators in the intestines and changes in the levels of biological markers in the kidneys (Dublineau et al. 2014). Others have investigated the ingestion of chronic low-dose cesium 137 and natural uranium in rats and, more recently, strontium 90 in mouse (Goudarzi et al. 2016; Grison et al. 2012, 2013). They pointed out specific metabolite fingerprint using a metabolomics approach. Metabolomics is a post-genomic approach which allows an exhaustive analysis of all measured metabolites in a biological matrix, reflecting the biological phenotype. Unfortunately, few studies used metabolomics in the field of low doses ionizing radiations and, more particularly, in low dose radionuclides intake (i.e. less than 10 publications since 2012). Therefore, the lack of scientific data justifies following this topic. In our previous studies (Grison et al. 2012, 2013), metabolomics analysis performed in rats chronically contaminated with either natural uranium (40 mg L^−1^) or cesium-137 (6500 Bq L^−1^) in drinking water showed for the first time the relevance of metabolomics in the field of chronic low dose radiotoxicology. Indeed, unlike other analytical techniques, metabolomics provided a discriminant fingerprint from urine of the contaminated group. These results open new perspectives but have to be validated with other studies including new cohorts, radionuclide dose–response and kinetic effect before concluding about the ability of metabolomics to cover the low dose ranges biological effect of radionuclide intake.

The objective of this study is to determine both the uranium concentration and the time required to observe a metabolic disruption in rats using a metabolomics approach. To investigate the dose–effect pattern of low-dose chronic exposure and the metabolomic changes induced, we used a toxicologically sensitive postnatal rat model, sub- and chronically contaminated with natural uranium in drinking water, from birth to adulthood. The environmental conditions of population exposure through drinking water (UNSCEAR 2008) were reproduced using uranium concentrations ranging from the threshold set by the WHO drinking-water guideline for uranium (0.03 mg L^−1^) (Frisbie et al. 2013) to the triple of the maximum environmental concentration measured in Finnish groundwater (12.4 mg L^−1^) (Salonen 1994).

## Materials and methods

### Animals and contamination procedure

All experimental procedures were approved by the Animal Care Committee of the Institute of Radioprotection and Nuclear Safety (IRSN) and complied with French regulations for animal experimentation (Ministry of Agriculture Act No. 87-848, October 19, 1987, modified May 20, 2001).

Sprague–Dawley rats, 12 weeks old and 16 days pregnant, were obtained from Charles River Laboratories (L’Arbresle, France). They were housed individually and maintained in a 12 h light/12 h dark cycle (regular cycle) at 21 °C and 50 % humidity, with access ad libitum to a standard rodent pellet diet and water. After weaning, female offspring and mothers were euthanized. Male offspring were housed, each paired with a rat from a different mother (assigned by randomization).

Rats were contaminated through their drinking water (Supplemental Fig. 1): natural uranium (NU) in a solution of uranyl nitrate hexahydrate (UO_2_(NO_3_)_2_,6H_2_O) was prepared to four final uranium concentrations of 40 (E dose); 1.5 (D dose); 0.15 (C dose) and 0.015 mg L^−1^ (B dose) (daily uranium intake: 1; 0.04; 0.004 and 0.0004 mg/rat/day) and dissolved in mineral water. These doses ranged from triple the highest uranium concentration of 12.4 mg L^−1^ found naturally, in well water in Finland (Salonen 1994), to half of the WHO 2011 drinking-water guideline for uranium, defined as equal to 0.030 mg L^−1^ (Frisbie et al. 2013). Natural uranium (Olympic) was obtained from CERCA (Pierrelatte, France). Control animals drank uncontaminated mineral water (A dose). The specific activity of the NU was 2.42 × 10^4^ Bq g^−1^, and its isotopic composition was ^238^U = 99.307 %, ^235^U = 0.688 %, and ^234^U = 0.005 %. Mothers of the offspring in the treated group were also exposed to NU through drinking water from birth to euthanasia (mothers of the control rats received uncontaminated mineral water). Until weaning, offspring were theoretically contaminated by mother’s milk [human offspring receive approximately 5 % of the mother’s daily dose (Wappelhorst et al. 2002)]. We monitored the food and water intake of both groups weekly throughout the contamination period.

### Collection of organs and biofluids

When rats were 3, 6 and 9 months old, they were placed in metabolic cages (in the morning, one per cage), with access to a rodent ground pellet diet and water (contaminated or not) ad libitum. Urine was collected twice a day for 48 h, with sodium azide (0.01 %) added to prevent bacterial growth (Griffin et al. 2001), and refrigerated at +4 °C. All samples for each rat were pooled, mixed, and centrifuged; supernatants were frozen at −80 °C. Rats were then moved back to conventional cages (one per cage) with food and drink ad libitum until the evening to reduce stress. At 9 months old, rats were euthanized. To control the diet cycle, food was removed in the evening until the next morning. Five hours before euthanasia, around 12 g of standard rodent pellet food was added directly to each cage to normalize food intake for all rats. Four hours later, rats were anesthetized by inhalation of 5 % isoflurane (Abbot France, Rungis, France) before an intracardiac puncture to collect blood, in heparinized tubes. Whole blood was centrifuged (5000 rpm) and plasma supernatants were immediately frozen at −80 °C. Kidneys were dissected on ice, weighed, deep-frozen in liquid nitrogen, and stored at −80 °C until uranium quantification.

### Biological and uranium analyses

#### Measurement of biochemical panel of markers

An automated spectrometric system (Konelab 20 from Thermo Electron Corporation, Cergy-Pontoise, France) was used for biochemical measurements of thawed urine samples, with the manufacturer’s biological chemistry reagents and protocols. The markers measured in urine included amylase, calcium, uric acid, creatinine, glucose, phosphorus, total proteins and urea.

#### Measurement of natural uranium organ contamination

Samples were mineralized (Ejnik et al. 2000) and analyzed for their uranium content by ICP-MS (XSERIE 2, Thermoelectron, France). A multielement standard solution (Analab, France) was used to optimize experimental conditions and apparatus parameters to obtain the best signal/noise ratio for ^238^U. In all solutions likely to be analyzed (biological samples or calibration solutions), bismuth 209 was added as an internal standard at 1 μg L^−1^. Six standard solutions for the calibration curve (0, 0.005, 0.01, 0.1, 0.5, and 1 μg L^−1^) were freshly prepared by dilution of a standard solution at 10 mg L^−1^ in 2 % nitric acid (NORMATOM for trace metal analysis, VWR Prolabo). A linear relation—count number (^i^U) = f([^i^U])—was calculated for each isotope, i = [235; 238] with [^i^U] equal to the isotope concentration in μg L^−1^. Isotopy and dosage reliability were regularly verified with standard solutions (6 quality controls at different concentrations and isotopy distributed throughout the analysis). Blank samples were run every five samples to check the stability of the background and to prevent potential contamination. For ^238^U, the detection and quantification limits were respectively 0.5 10^−3^ and 1.5 10^−3^ µg L^−1^, and for ^235^U, 0.01 10^−3^ and 0.03 10^−3^µg L^−1^. The limits for ^238^U were applied to total uranium.

#### Renal dose estimation (9 months postnatal)

The radiation yield and energy emitted during the nuclear transformation of the isotopes forming NU come from ICRP (1983). Alpha particles and Auger and internal conversion electrons were assumed to be locally absorbed in the target organ. Photons were transported with the code MCNPX in a voxel phantom of an adult male rat from the same strain. Given the preponderant concentration of NU in kidneys and the small fraction of energy emitted as penetrating radiation, kidney irradiation by NU from the rest of the body was ignored because negligible. The absorbed dose rate to the kidney was thus determined at 9 months of age based on the kidney concentration of NU and kidney mass (Table 1, panel A) and assuming a homogeneous concentration of NU throughout the entire 9 months.

**Table 1:** Biological and uranium analyses

	Total body weight (g)	Kidney weight (g)	Ratio kidney/total body (%)	Uranium concentrations in kidney (ng U g^−1^)					
Panel A									
Control (20)	577 ± 9	1/77 ± 0.03	0.3	6.3 ± 0.6					
NU 0.015 mg L^−1^ (20)	572 ± 14	1.71 ± 0.08	0.3	6.5 ± 0.6					
NU 0.15 mg L^−1^ (20)	559 ± 8	1.71 ± 0.04	0.3	8.7 ± 0.8*					
NU 1.5 mg L^−1^ (20)	593 ± 15	1.72 ± 0.05	0.3	16 ± 2***					
NU 40 mg L^−1^ (20)	561 ± 13	1.64* ± 0.03	0.3	268 ± 34***					

	Urine volume (g)	Amylase (U)	Calcium (mmol)	Uric acid (µmol)	Creatinine (µmol)	Glucose (mmol)	Phosphorus (mmol)	Total proteins (g)	Urea (mmol)
Panel B									
3 months									
Control (20)	28.40 ± 1.20	174.05 ± 14.48	0.05 ± 0.01	47.34 ± 6.14	222.87 ± 6.93	0.08 ± 0.01	0.52 ± 0.10	0.06 ± 0.01	21.33 ± 1.15
NU 0.015 mg L^−1^ (20)	25.25 ± 1.37	147.04 ± 16.10	0.04 ± 0.00*	43.71 ± 6.43	215.17 ± 6.56	0.07 ± 0.01	0.50 ± 0.08	0.10 ± 0.05	17.17 ± 1.0**
NU 0.15 mg L^−1^ (20)	27.50 ± 2.59	135.51 ± 20.13	0.04 ± 0.01	93.64 ± 55.89	209.21 ± 7.83	0.08 ± 0.01	0.62 ± 0.14	0.13 ± 0.06	17.94 ± 0.82*
NU 1.5 mg L^−1^ (20)	24.45 ± 1.22*	159.39 ± 19.90	0.04 ± 0	37.96 ± 3.68	220.05 ± 8.46	0.07 ± 0.01	0.46 ± 0.07	0.05 ± 0.00	18.65 ± 0.69
NU 40 mg L^−1^ (20)	25.75 ± 1.37	156.94 ± 15.48	0.039 ± 0.00	35.82 ± 1.22	220.32 ± 5.75	0.09 ± 0.02	0.52 ± 0.06	0.06 ± 0.01	18.29 ± 0.47*
6 months									
Control (20)	31.01 ± 1.29	217.45 ± 8.63	0.08 ± 0.01	40.30 ± 1.23	279.18 ± 8.79	0.06 ± 0.00	0.44 ± 0.05	0.08 ± 0.01	26.07 ± 0.76
NU 0.015 mg L^−1^ (20)	26.50 ± 1.2*	193.63 ± 8.19	0.06* ± 0.01	41.30 ± 2.35	275.27 ± 8.45	0.06 ± 0.01	0.38 ± 0.04	0.06 ± 0.00**	23.47 ± 0.73*
NU 0.15 mg L^−1^ (20)	26.06 ± 1.14**	206.52 ± 9.62	0.05** ± 0.01	39.11 ± 1.22	255.79 ± 8.27	0.06 ± 0.00	0.49 ± 0.04	0.07 ± 0.01	22.00 ± 0.73***
NU 1.5 mg L^−1^ (20)	28.22 ± 1.65	226.06 ± 15.26	0.06 ± 0.01	38.23 ± 1.59	279.61 ± 11.87	0.06 ± 0.00	0.45 ± 0.06	0.07 ± 0.00	24.93 ± 0.95
NU 40 mg L^−1^ (20)	26.03 ± 1.66*	206.85 ± 8.24	0.06** ± 0.01	39.80 ± 1.31	269.18 ± 8.62	0.06 ± 0.00	0.37 ± 0.06	0.06 ± 0.00**	22.85 ± 0.55***
9 months									
Control (20)	31.17 ± 1.86	240.23 ± 10.02	0.07 ± 0.01	39.71 ± 1.53	292.78 ± 9.89	0.06 ± 0.01	0.66 ± 0.05	0.12 ± 0.02	26.33 ± 0.78
NU 0.015 mg L^−1^ (20)	28.41 ± 2.00	216.90 ± 8.92	0.06 ± 0.01	40.67 ± 2.41	289.46 ± 7.88	0.06 ± 0.00	0.64 ± 0.04	0.08 ± 0.01*	24.49 ± 0.77
NU 0.15 mg L^−1^ (20)	24.37 ± 0.90**	240.24 ± 14.90	0.06 ± 0.01	40.00 ± 1.49	277.05 ± 4.84	0.06 ± 0.00	0.65 ± 0.05	0.10 ± 0.02	22.53 ± 0.46***
NU 1.5 mg L^−1^ (20)	27.30 ± 1.76	258.68 ± 15.75	0.06 ± 0.01	39.29 ± 1.73	301.15 ± 9.01	0.05 ± 0.00	0.72 ± 0.04	0.10 ± 0.01	26.13 ± 0.83
NU 40 mg L^−1^ (20)	23.86 ± 1.21**	229.77 ± 12.97	0.06 ± 0.01	39.70 ± 1.28	286.41 ± 9.01	0.05 ± 0.00	0.56 ± 0.04	0.07 ± 0.01**	23.62 ± 0.82*

*Panel A* Mean ± SEM of (i) whole-body weight, kidney weight and ratio of kidney to whole-body weight in each group, and (ii) uranium concentration in kidneys after 9 months of chronic radionuclide ingestion through drinking water (0.015, 0.15, 1.5 and 40 mg L^−1^)

*Panel B* Mean ± SEM of (i) 48-h urine collection, and (ii) urine proteins, carbohydrates, ions and other metabolites in control and contaminated (NU) groups after 3, 6 and 9 months of chronic radionuclide ingestion through drinking water (0.015, 0.15, 1.5 and 40 mg L^−1^)

The number of rats for each measurement is indicated in parentheses

Results are significantly different for: * *P* < 0.05; ** *P* < 0.01; *** *P* < 0.001

#### Statistical analysis

Values of the biochemical and clinical parameters were reported as means ± standard errors of the means (SEM). The control and contaminated groups were compared with Student’s t test in normal populations or the rank sum test in non-normal populations. Statistical significance was defined by a P value less than 0.05. Statistical analyses were performed with SigmaStat statistical software (SPSS, Paris, France).

### Metabolomic analyses

#### Sample preparation and LC–MS analyses

Urine samples were diluted with ultrapure water (1:4, v/v) before analysis. After centrifugation for 15 min at 11,000 rpm and 4 °C, 100 µL of thawed urine was mixed and shaken for 1 min with 300 µL of LC–MS (liquid chromatography-mass spectrometry) grade water. After a second centrifugation for 5 min at 3000 rpm, 50 µL of supernatant was transferred into HPLC vials and stored at −80 °C prior to analysis.

To ensure reproducible and robust data acquisition (Dunn et al. 2011), the 300 samples (5 doses at each of 3 time points for each of the 20 rats) were analyzed as 5 smaller analytical batches of 60 samples. Each of the randomized batches followed a typical injection order: a blank sample (LC–MS grade water) was injected four times at the start, then a pooled sample (a mixture of all samples) was injected ten times; thereafter every fifth injection was inserted a pool quality control (QC) sample (made up with small aliquot of the samples of all series) throughout the batch series.

The samples were analyzed on an Agilent 1200 RRLC coupled to a Bruker micrOTOF ESI-hybrid quadrupole-time of flight mass spectrometer (Wissembourg, France), both devices driven by the Compass 1.3 SR 1 for micrOTOF/maXis software (Bruker Daltonics). The LC conditions were: injection volume, 5 µL; autosampler temperature, 4 °C; column type, EC 100/2 Nucleodur C18 pyramid (Macherey–Nagel, Les Ulis, France); particle size, 1.8 μm; column length, 100 mm; column internal diameter, 2 mm; column temperature, 40 °C; solvent flow, 0.4 mL min^−1^ (solvent A: 95 % water, 5 % acetonitrile, 0.1 % formic acid, and solvent B: 95 % acetonitrile, 5 % water, 0.1 % formic acid); and gradient, 3 % B for 1 min, 3–30 % B for 7 min, 30–95 % B for 1 min, 95 % B for 1 min, 95–3 % B for 1 min, and 3 % B for 4 min (running time: 15 min). The MS conditions were as follows: acquisition mode, positive electrospray ionization (ESI+) and full scan 50–1500 *m*/*z*; capillary voltage, 4.5 kV; capillary temperature, 200 °C; cone voltage, 40 V; drying gas flow, 9.5 L min^−1^; and nebulizing gas pressure (nitrogen), 2.9 bar; calibration, internal with injection of sodium formate acetate at the beginning of every run.

#### Data preprocessing and filtering

LC–MS raw data were exported to “.cdf” file format with the manufacturer’s DataAnalysis software (Bruker, Wissembourg, France) and preprocessed with the freely available XCMS software, including the CAMERA script (Smith et al. 2006). Peak picking was performed with the ‘centWave’ method (‘peakwidth’ parameter reduced to 3–15 s to fit UPLC performances, and ‘snthresh’ to 5 to detect more peaks), retention time correction with the obiwarp method (‘profStep’ reduced to 0.1 *m*/*z* as recommended for QTOF mass spectrometers), peak grouping with the ‘bw’ and ‘mzwidth’ parameters reduced respectively to 5 and 0.025, and gap filling with the default parameters.

Raw data quality was checked by principal component analysis (PCA), with SIMCA-P + 12.0 software (Umetrics, Umeå, Sweden), both for each single batch and for all batches together. The presence of any individual outlier was ruled out. Signal drift over time was quite weak within batches and unsurprisingly higher between them. Signals were corrected for both drifts with the Van der Kloet algorithm (a linear model) (van der Kloet et al. 2009) embedded into an R script (generous gift from Jean-François Martin, INRA AXIOM METATOUL, Toulouse, France).

Data normalization was followed by a filtering step based on the coefficient of variation of variable intensity in the pooled sample (cutoff set at 20 %), which reduced the number of variables from 2583 to 1736 (67 % remaining). The removal of variables detected before 24 s (mostly from the calibration solution) left the dataset with 1718 variables. Finally, the data were log10-transformed and Pareto-scaled before the statistical analysis (Martin et al. 2015).

#### Statistical analyses

Multivariate statistical analyses were performed with either SIMCA-P + 12.0 software (Umetrics, Umeå, Sweden) or R packages (base, pRoc, HDMD). Partial least squares discriminant analysis (PLS-DA) models were validated by CV-ANOVA (threshold for significance set at 0.05) and by permutation tests (200 permutations, test passed for R2Y and Q2Y value decreased, below zero for Q2Y one). Three “blocs” of statistical analyses and features selection were applied to the preprocessed and filtered matrix (Supplemental Fig. 2), aiming at (1) compare the present cohort to a previous one, (2) distinguish the time point effect from the dose effect, and (3) investigate finely the dose effect.

##### Discrimination between controls and rats contaminated at the E-dose (40 mg L^−1^) after 9 months

The aim was to compare the results obtained in the present cohort with those obtained in our previous cohort in 2013 (Grison et al. 2013), to check its reproducibility. PLS-DA was performed on a subset of the filtered dataset including the control rats (n = 20) and the E-dose contaminated rats (n = 19; 1 sample lost) at 9 months. The most discriminatory metabolites were then selected, according to their ‘variable importance in projection’ (VIP) score of the PLS algorithm. The top 95 of these, having the highest VIP score, were retained for comparability with the number of variables selected in our previous cohort (and was again equivalent to 1.5 < VIP score < 2.4) (Grison et al. 2013).

##### Separate analyses of time-point and dose effects

At this second step of our statistical analysis we performed ANOVA-PCA (APCA) on the overall dataset. Indeed, we investigated two contamination factors simultaneously: the duration (time-point effect) and the level (dose effect). As for the multivariate data, depending on several factors, we used APCA, which is a powerful tool to evaluate significant factors of an experimental design and select the principal features associated with each factor (Harrington et al. 2005; Zwanenburg et al. 2011; Climaco Pinto et al. 2008). Briefly, it decomposed the original matrix into effect matrices (time-point effect, dose effect, dose × time-point interaction effect) and a matrix containing the residual error. Each effect matrix was calculated as the average of the variables at each level of the corresponding factor, and the residual matrix as the difference between the original matrix and the sum of the effect matrices. Adding the effect matrices to the residual matrices produced two separate matrices: the “time-point matrix”, which was the sum of the time-point factor and the residual error, and the “dose matrix”, the sum of the dose factor, the dose_*_time-point interaction factor, and the residual error. PCA was then performed on each matrix to select the features most associated with each effect (cut-off was set at 2 standard deviations for absolute value of loading, which corresponds to the level of significance used for a standard normal distribution). As Harrington et al. point out, the dominant factor should appear on the first component of the PCA while the other components explain the residual errors. Nonetheless, the biological variation in metabolomics data may be greater than the experimental variation, so that the significant factor might be found on the second or any subsequent PCA component. This was the case for the dose matrix. To overcome this weakness, we used the method for selective reduction of residual variability proposed by Climaco Pinto et al. (2008) to identify features associated with the dose effect. Finally, to check the robustness of each feature selection (i.e., for time-point effect and for dose effect), 3 PLS-DA models were built separately: one based on the time-point effect matrix and two based on the dose effect matrix. The first PLS-DA was built on all classes, i.e., the control group and all four contamination doses, one low (E: 40 mg L^−1^ of drinking water) and three very low (B: 0.015 mg L^−1^; C: 0.15 mg L^−1^; D: 1.5 mg L^−1^), while the second PLS-DA model was built on all classes except that receiving the E dose, i.e., on the control group and the three very low-dose groups.

##### Dose effect according to contamination duration and selection of the most discriminant features

The third and last step of our statistical analysis aimed at refining our investigation of the dose effect (without the confounding factor of the duration in itself, but keeping the dose along with the dose × time-point interaction factors) and was based on the features selected in APCA (supplemental Fig. 2). 16 PLS-DAs were performed on the dose effect matrix to search for any discrimination between the control group and each contaminated group, i.e. B-dose (0.015 mg L^−1^), C-dose (0.15 mg L^−1^), D-dose (1.5 mg L^−1^) and E-dose (40 mg L^−1^) contaminated rats, separately (main characteristics of these PLS-DA models are listed in Table 2, panel B). The existence of such discrimination was checked regardless of the time-points, or after 3, 6 and 9 months of contamination. For six out of the sixteen models, the most discriminatory features were selected by their VIP score (set at >1.2, in accordance with our previous studies): control vs. B-dose (0.015 mg L^−1^) contaminated rats at all time points (model 1, Table 2, panel B), control vs. C-dose (0.15 mg L^−1^) contaminated rats at all time points (model 5, Table 2, panel B), control vs. D-dose contaminated rats at all time points (model 9, Table 2, panel B), control vs. E-dose (40 mg L^−1^) contaminated rats after 3 months of contamination (model 14, Table 2, panel B), control vs. E-dose (40 mg L^−1^) contaminated rats after 6 months of contamination (model 15, Table 2, panel B), and control vs. E-dose (40 mg L^−1^) contaminated rats after 9 months of contamination (model 16, Table 2, panel B). Using Venn diagrams (Oliveros 2007), the lists of selected features were compared to each other to check for common features: on one hand, we compared the selected features for the three very low-dose groups (models 1, 5 and 9) and, on the other, the selected features for the E-dose (40 mg L^−1^) group after 3, 6 and 9 months of contamination (models 14, 15 and 16).

**Table 2:** Characteristics of PLS-DA models

Cohort (number of variables in the matrix)	Components number	Observations number	R2Y (%)	Q2Y (%)	CV-ANOVA	
Panel A						
Grison et al. (2013) (1376)	2	20	91.9	55.2	9.40e−03	
Grison et al. (2013) (95)	2	20	88.9	74.2	4.60e−04	
Present article (1718)	3	39	95.5	75.2	1.70e−06	
Present article (95)	2	39	88.0	80.2	7.77e−12	

Discrimination	Model	Components number	Observations number	R2Y (%)	Q2Y (%)	CV-ANOVA
Panel B						
A vs. B						
All time points	1	2	120	26.9	16.7	2.23e−05
3 month	2	0	40	–	–	–
6 month	3	0	40	–	–	–
9 month	4	0	40	–	–	–
A vs C						
All time points	5	2	120	57.3	40.3	6.42e−10
3 month	6	0	40	–	–	–
6 month	7	2	40	46.6	16.7	0.0340864
9 month	8	2	40	51.6	30.3	0.00127167
A vs D						
All time points	9	3	120	53.2	33.3	6.04e−05
3 month	10	2	40	61.8	33.5	0.0105006
6 month	11	0	40	–	–	–
9 month	12	0	40	–	–	–
A vs E						
All time points	13	3	117	76.7	69.6	2.44e−23
3 month	14	3	39	81.4	58.3	0.00698012
6 month	15	2	39	81.5	64.4	6.87e−07
9 month	16	3	39	83.7	70.3	4.00e−05

*Panel A* Models discriminating the control rats from those contaminated for 9 months at the dose 40 mg L^−1^ in the present study and in our previous proof-of-principle study

*Panel B* Analyses performed on the “dose matrix” after feature selection (126 variables) to investigate the dose effect; models are discriminating the control from the contaminated rats for each dose (dose B: 0.015 mg L^−1^; dose C: 0.15 mg L^−1^; dose D: 1.5 mg L^−1^; dose E: 40 mg L^−1^) after 3, 6 and 9 months of contamination and all time-points together

Finally, the features thus selected were used to estimate composite scores able to distinguish each dose from the others to the possible extent. Four scores were calculated: one for all time points and one for each single time point (3, 6 and 9 months). These were built as linear combinations of the features and loadings estimated in the PLS-DA regression predicting the discrimination between the control group and each contaminated group. The distribution of the composite score was graphically represented with boxplots across each experimental group, both including all three time points and separately (at 3, 6 and 9 months). To test the robustness of the predictions, Receiver Operating Characteristic curves (ROC) and Area Under the Curve (AUC) were computed between the control and each contaminated groups, for all time points, and separately (at 3, 6 and 9 months).

#### Metabolite identification

For the most discriminant features, a tentative annotation was performed with MZedDB (http://www.maltese.dbs.aber.ac.uk:8888/hrmet/index.html) (Draper et al. 2009) from the chemical formulas generated from the accurately measured masses (accuracy <10 ppm) and isotopic patterns, calculated with Bruker DataAnalysis software. According to the putative identifications returned, 20 standard molecules were purchased. l-lysine (L5501-1G, CAS 56-87-1), N6-methyl-l-lysine hydrochloride (04685-100MG, CAS 7622-29-9), (-)-epinephrine (E4250-1G, CAS 51-43-4), 4-pyridoxic acid (P9630-25MG, CAS 82-82-6), 3-methoxytyramine hydrochloride (65390-250MG, CAS 1477-68-5), methyl hippurate (S750115-100MG, CAS 1205-08-9), suberic acid (S5200-5G, CAS 505-48-6), 4-methylcathecol (M34200-5G, 452-86-8), 2-phenylethanol (77861-250ML, 60-12-8), l-alpha-lysophosphatidylcholine type I from egg yolk (L4129-25MG, CAS 9008-30-4), sebacic acid (283258-5G, CAS 111-20-6), and linoleic acid (L1376-500MG, CAS 60-33-3) were purchased from Sigma-Aldrich (L’Isle D’Abeau Chesnes, 38297 St. Quentin Fallavier, France). 1-Methylnicotinamide (3-carbamoyl-1-methylpyridinium chloride, M0375-5G, CAS 1005-24-9), *N*-tigloylglycine (T1260-100MG, CAS 35842-45-6), 2-hydroxy-*n*-octanoic acid (H0592-5G, CAS 617-73-2), and *trans*-2-octenoic acid (O0004-10ML, CAS 1871-67-6) were purchased from TCI Europe (Boerenveldseweg 6, Haven 1063, 2070 Zwijndrecht, Belgium). N1-Methyl-2-pyridone-5-carboxamide (TLCN-0621-10MG, CAS 701-44-0), rac *N*-formiminoglutamic acid (TRC-F735500-100MG, 816-90-0), and 3-methylcrotonyl Glycine (TRC-M294540-50MG, CAS 33008-07-0) were purchased from LGC Standards (6 rue Alfred Kastler, BP 83076, 67123 Molsheim cedex, France). 4-Hydroxyphenylacetylglycine (EN300-65253-1G, CAS 28116-23-6) was purchased from Enamine Ltd (Vestienas iela 2b, 1035 Riga, Latvia). All standard molecules were solubilized in water/acetonitrile (50:50), except linoleic acid, which was solubilized in chloroform, and injected at a concentration of 10 mg L^−1^. Experimental samples displaying the highest intensity for each discriminant feature were selected, and both standard molecules and experimental samples underwent MS and MS/MS experiments. MS conditions were the same as for initial acquisitions; MS/MS parameters were: isolation width, 0.8; cell (collision) energy, 15, 20, 25 and 30 eV; acquisition factor, 1. Full MS and MS/MS spectra were compared between standard molecules, experimental samples and spectral databases (mainly HMDB, Metlin and MassBank) for final metabolite identification.

## Results

### Effect of chronic low-dose exposure on biochemical and clinical parameters

Both consumption of drinking water and food and body weight gain were monitored once a week throughout the experiment and did not differ between control and contaminated rats. The whole body and one kidney (on the same side for each animal) were weighed at euthanasia for all rats and did not differ significantly between groups. The volume of urine collected during the 48-h period before sacrifice (25 mL on average) and the relative weight of the kidneys were statistically homogeneous (Table 1, panels A, B). Several biochemical markers were also assessed in urine samples, including ions and kidney markers. Except for slight (but within the physiological range) modifications in some concentrations in the contaminated rats, no significant difference between the groups was observed (Table 1, panel B). These results confirm our previous observations for the E-dose (40 mg L^−1^) after 9 months of contamination (Grison et al. 2013). Our data also confirm that uranium accumulates in the kidney at a rate equivalent to that we reported previously. Furthermore, our measurements of the rate of uranium accumulation in the kidneys were correlated to the uranium concentrations in the drinking water. Thus, for example, at 9 months of age and for the E-dose of 40 mg L^−1^, the absorbed dose rate in the kidneys of the contaminated rats was estimated at 5.4 × 10^−7^ Gy day^−1^, corresponding to a maximum dose absorbed by the kidneys at sacrifice as low as 0.15 mGy (assuming a constant intake of NU throughout the entire 9-month period).

### Discrimination between control and E-dose (40 mg L^−1^) contaminated rats after 9 months

**Fig. 1:**
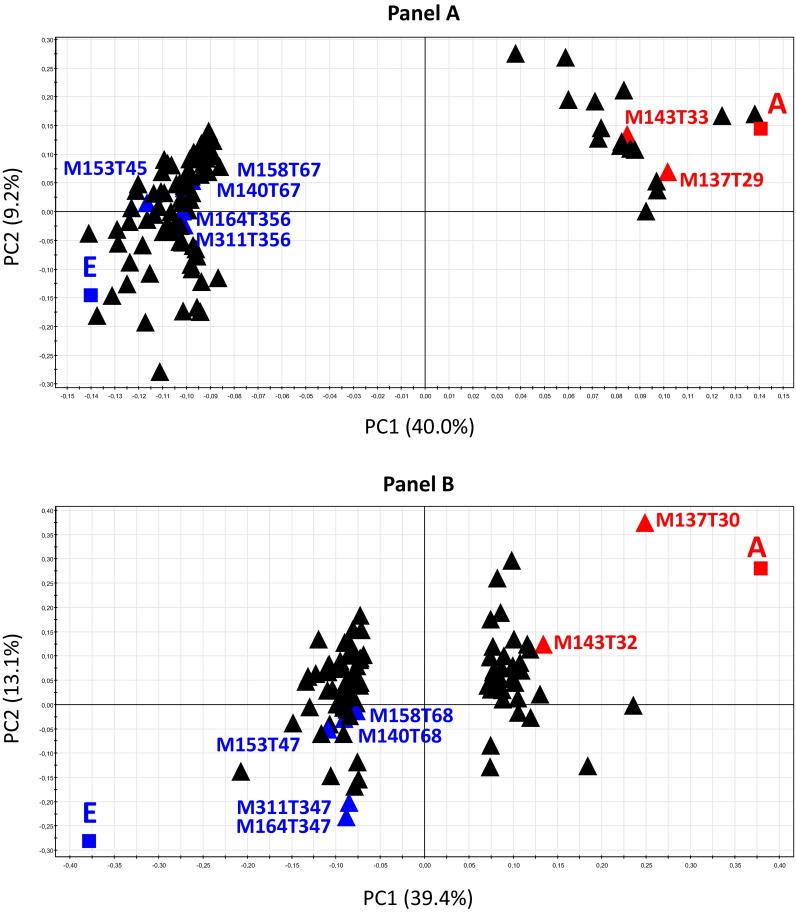
Comparison between the present and a previous cohort. Discriminations were performed between the control rats (A group) and the rats contaminated at the dose 40 mg L^−1^ for 9 months (E group). Loading scatter plots from the partial least-square discriminant analysis (PLS-DA) were based on the 95 most discriminant features. **a** Present cohort; variable selection was performed on the preprocessed and filtered matrix (1718 variables; VIP scores >2.4). **b** Cohort from Grison et al. (2013); variable selection was performed on the preprocessed and filtered matrix (1376 variables; VIP scores >1.8)

The PLS-DA of the control and E-dose (40 mg L^−1^) contaminated rats at 9 months produced a highly validated and robust model (CV-ANOVA = 1.70e−06, R2Y = 96 %, Q2Y = 75 %) that very clearly discriminated between the groups (Table 2, panel A). This intergroup variation was largely captured by the first component (40 % of total variance) (Fig. 1a). The selection of the 95 most discriminant features enhanced the CV-ANOVA (7.77e−12) and the Q2Y (80 %) values (Table 2, panel A). These results reproduced our previous findings and confirmed that untargeted metabolomics in urine is an appropriate approach for exploring low-dose uranium radiotoxicology. The characteristics of the PLS-DA models were quite similar (Grison et al. 2013) between the proof-of-principle (Grison et al. 2013) and the present validation cohorts, with an increase in CV-ANOVA and Q2Y values that appear to be explained by the doubling of the cohort size (Table 2, panel A). When we looked specifically at the LC–MS ID of the 95 most discriminant features in both cohorts (Fig. 1), we found that 7 of those were common. In particular, a previously identified metabolite, N1-methylnicotinamide (*m*/*z* 137), ranked 13th according to its VIP score in the validation cohort.

### Separate analyses of time-point effect and dose effect

PCA of the time-point matrix showed that component 2 characterized the time-point effect, mostly represented by 121 variables (2SD corresponded to absolute value of loadings >0.045). PLS-DA of the selected features showed a validated, robust discrimination between the samples collected at 3, 6 and 9 months (CV-ANOVA <0.001, R2Y = 90 %, Q2Y = 87 %) (data not shown). This strong effect is unsurprising and probably associated more with the aging of the rats than with the duration of their contamination.

**Fig. 2:**
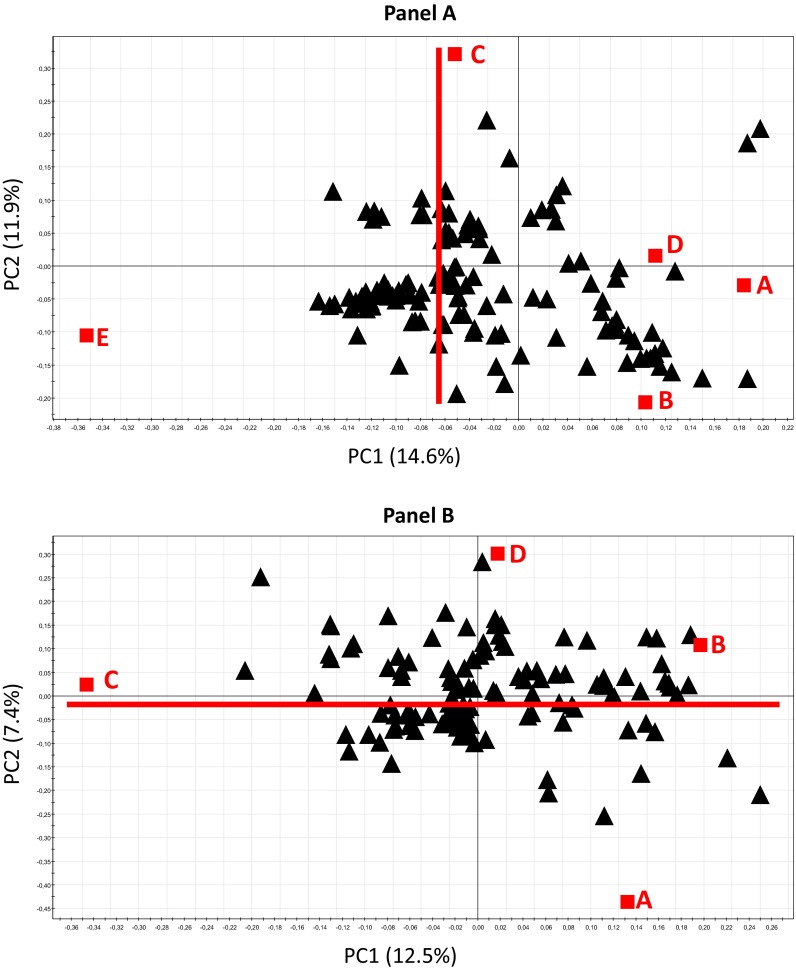
Partial least-square discriminant analysis (PLS-DA) performed on the “dose matrix” after feature selection (126 variables); classes were: *A*,control; *B* contamination dose of 0.015 mg L^−1^;* C* 0.15 mg L^−1^;* D* 1.5 mg L^−1^;* E* 40 mg L^−1^. **a** Loading* scatter plot* from the model built on all experimental doses. **b** Loading* scatter plot* from the model built on all experimental doses but the E one

PCA of the dose matrix showed that components 11 and 12 characterized the dose effect, with 126 variables highly associated with it (2SD corresponded to absolute value of loadings >0.040). PLS-DA of the selected features for all experimental doses (Fig. 2a) was validated (CV-ANOVA = 1.1e−20, R2Y = 19 %, Q2Y = 14 %) and showed a mild dose effect, but a clear shift between the low and the very low doses on the first principal component (describing 14.6 % of the total variance). The second PLS-DA of the selected features for all but the E dose (Fig. 2b) was also validated (CV-ANOVA = 4.0e−12, R2Y = 15 %, Q2Y = 9.5 %); it too showed a mild dose effect, but marked discrimination between the control and contaminated groups for the second principal component (describing 7.4 % of the total variance). Both PLS-DAs showed good discrimination between the control and C-dose (0.15 mg L^−1^) contaminated rats (on PC2 and PC1 in the first and second models, respectively). These results highlighted a difference between the low (E: 40 mg L^−1^) and very low (B: 0.015 mg L^−1^, C: 0.15 mg L^−1^ and D: 1.5 mg L^−1^) doses; we therefore analyze their data separately.

### Dose effect according to contamination duration and selection of most discriminant features

Our first observation regarding the dose effect was that the higher was the dose, the greater was the robustness of the discrimination. This was assessed by the R2Y and Q2Y values of the models built on all time points (models 1, 5, 9 and 13, Table 2, panel B), which were all validated according to their CV-ANOVA (p values ranging from 2.23e−05 to 2.44e−23 for the B and E doses, respectively). Indeed, when increasing the dose of contamination, the observed variance (R2Y value) of the model increased (27, 57, 53 and 77 %, for the B (0.015 mg L^−1^), C (0.15 mg L^−1^), D (1.5 mg L^−1^) and E (40 mg L^−1^) doses, respectively), as did the predicted variance (Q2Y value) (17, 40, 33 and 70 % for the B (0.015 mg L^−1^), C (0.15 mg L^−1^), D (1.5 mg L^−1^) and E (40 mg L^−1^) doses, respectively).

**Fig. 3:**
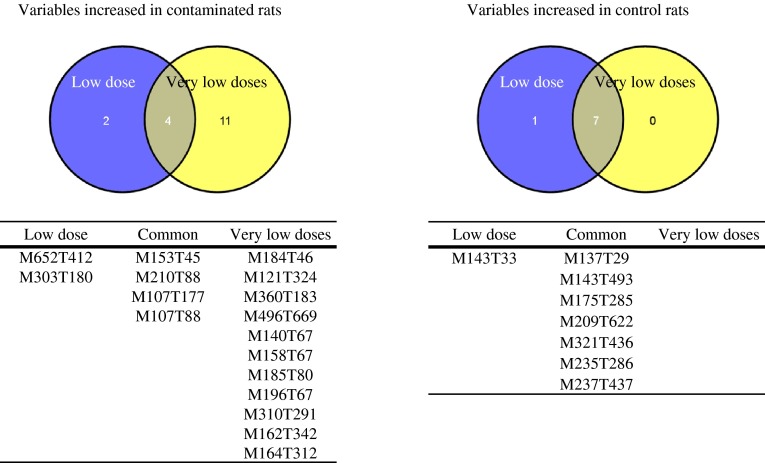
Selection of the most robust features associated with low- and very low-dose exposure to natural uranium. Using Venn diagrams, the 14 features associated to the E-dose contamination whatever the duration of the contamination (“Low dose” group) were compared to the 22 features associated to any of the other doses of contamination (B, C and D) when pooling all time points together (“Very low dose” group)

We thereafter focused on the effect of the contamination duration for the E-dose. We observed that the discrimination between control and contaminated rats for this low dose started as a strong trend from 3 months of contamination onward (model 14, Table 2, panel B; R2Y = 81 %, Q2Y = 58 %) and increased with contamination duration after 6 months (model 15, Table 2, panel B; R2Y = 82 % and Q2Y = 64 %), and after 9 months (model 16, Table 2, panel B; R2Y = 84 % and Q2Y = 70 %). We selected the features most strongly associated with NU contamination after 3, 6 and 9 months (23, 34 and 29 features selected, respectively; data not shown), and compared the selection lists to each other to check for a set of features associated with NU contamination whatever the duration: 14 features were common to at least 2 contamination durations (Fig. 3, features in the “Low dose” group).

Thirdly, we scrutinized the effect of the contamination duration for the other lower doses. Except for the C-dose contamination, the data did not show results as easy to interpret as those obtained for the E-dose (0.15 mg). No supervised discrimination was achieved for the B-dose (0.015 mg) contamination whatever the duration (models 2, 3 and 4, Table 2, panel B). It remains unclear whether this result is explained by the too-low dose level or by too few observations, since discrimination was achieved on the model built with all time points (model 1, Table 2, panel B). For the C-dose (0.15 mg) contamination, a trend towards discrimination appeared after 6 months of contamination (model 7, Table 2, panel B; R2Y = 47 %, Q2Y = 17 %) and was stronger after 9 months (model 8, Table 2, panel B; R2Y = 52 %, Q2Y = 30 %). For the D-dose (1.5 mg) contamination, a trend was observed after 3 months (model 10, Table 2, panel B; RY2 = 62 %, Q2Y = 34 %), but was no longer observed after 6 or 9 months of contamination (models 11 and 12, Table 2, panel B, respectively). To smooth the inconstancy of the time effect, we then considered all the time points together to seek for discriminating features of contamination (models 1, 5 and 9, Table 2, panel B). There were 31 features highly responsible for discriminating the control group from the B-dose (0.015 mg L^−1^) group (model 1), 26 from the C-dose (0.15 mg L^−1^) group (model 5), and 28 from the D-dose (1.5 mg L^−1^) group (model 9). Among those features, 22 were common to at least 2 contamination doses (Fig. 3, features in the “Very low doses” group).

To achieve a robust feature selection, we compared the 14 features associated to the E-dose contamination whatever the duration (Fig. 3, group “Low dose”) to the 22 features associated to the other doses of contamination when pooling all time points together (Fig. 3, group “Very low doses”). Eleven features were common to both groups, 4 of which were increased in contaminated rats (Fig. 3, table on the left, middle column) and 7 were increased in control rats (Fig. 3, table on the right, middle column). When compared to control rats, 4 ROC-AUCs were computed for each of these 11 features the most associated with chronic low and very-low dose natural uranium contamination: (Supplemental Table 1). Of the 11 features, 10 had at least one ROC-AUC value >70 %, 6 of the 11 >80 %.

**Fig. 4:**
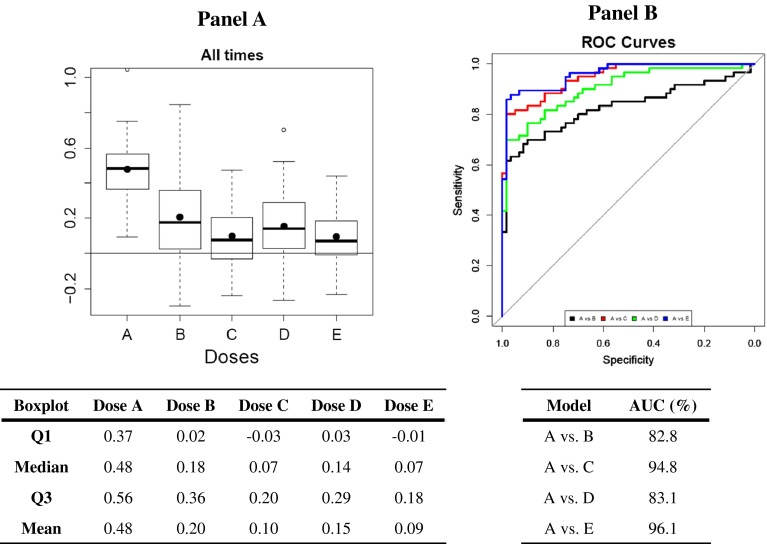
Composite score built for all time points with the 11 features most associated with exposure to natural uranium (*A* control;* B*,contamination dose of 0.015 mg L^−1^;* C* 0.15 mg L^−1^;* D* 1.5 mg L^−1^;* E* 40 mg L^−1^) **a** *Boxplots*. **b** ROC curves and area under the ROC curves (AUC)

Finally, we used these 11 most robust discriminant features to build four composite scores: one for all time points and one for each single time point (3, 6 and 9 months). The PLS-DA models computing the composite scores were estimated (R2Y = 19.2 % and Q2Y = 14.3 % for the one build at all time points) and validated (CV-ANOVA <0.001 and a highly convincing permutation test). As the boxplots show, composite scores discriminated well between the control and contaminated groups, both for all time points (Fig. 4a) and for each single time point (Supplemental Fig. 2). The ROC curves and their AUC further demonstrated the robustness of these composite scores (Fig. 4b; Supplemental Fig. 2). All ROC-AUC values were greater than 81.5 (the B dose after 3 months of contamination). The best composite scores were observed for the C (94.8 %) and E (96.1 %) doses for all time points. The composite score even predicted discrimination between the control and contaminated groups at almost 100 % (ROC-AUC = 99.2 %, Supplemental Fig. 2) for the E dose after 9 months of contamination.

### Identification of the most discriminant features associated with chronic low-dose contamination by natural uranium

We analyzed and tested 20 standard molecules for a match with the 11 top discriminant features common to all doses, and with the next 14. Five features were identified as existing metabolites: 3 among the 11 most discriminant features (N1-methylnicotinamide, N1-methyl-2-pyridone-5-carboxamide, and 4-hydroxyphenylacetylglycine) and 2 of the other 14 (l-alpha-lysophosphatidylcholine and 4-pyridoxic acid). Identifications were confirmed according to high resolution retention time, accurate mass of parent ion, molecular formula based upon accurate *m*/*z* and isotope pattern, accurate mass tandem mass spectrum and, when available, matches to spectral libraries (Supplemental Fig. 3–7). According to the newly proposed scoring system for reporting metabolite identification (Sumner et al. 2014), these molecules all had score values higher than 11, which is the value proposed for confident identification (when not taking spectral libraries into account).

N1-methylnicotinamide was detected as [M1+] (*m*/*z* 137.07) in both standard solution and experimental sample at 29–30 s. Fragmentation spectra were superimposable and showed a major fragment at *m*/*z* 94.06 (Supplemental Fig. 3). This metabolite was previously identified in our proof-of-principle study (Grison et al. 2013). Such a fragmentation spectrum was recorded in the Human Metabolome Database (HMDB00699).

N1-Methyl-2-pyridone-5-carboxamide was detected as [M + H] (*m*/*z* 153.06), along with its sodium adduct (*m*/*z* 175.05), in both the standard solutions and experimental samples at 50–53 s. Fragmentation spectra were superimposable and showed a major fragment at *m*/*z* 108.04 (Supplemental Fig. 4). No spectral data were available in databases.

4-Hydroxyphenylacetylglycine was detected as [M + H] (*m*/*z* 210.08), along with its sodium and potassium adducts (*m*/*z* 232.06 and 248.03, respectively), in both the standard solution and experimental sample at 123–126 s. Fragmentation spectra were superimposable and showed a major fragment at *m*/*z* 107.05, as recorded in the Human Metabolome Database (HMDB00735) (Supplemental Fig. 5). Both [M + H] and its fragment were in the top 11 discriminant features.

L-alpha-Lysophosphatidylcholine from egg yolk (with its usual fatty acid contents of approximately 66 % palmitic and 33 % stearic acids) was detected as [M + H] (*m*/*z* 496.34), along with its sodium adduct (*m*/*z* 518.32), in both the standard solution and experimental sample at 672–676 s. Both peaks had similar split shapes, corresponding to the isomerization of the molecule. Fragmentation spectra were superimposable and showed two major fragments at *m*/*z* 104.10 and 184.07, corresponding to choline and phosphorylcholine, respectively (Supplemental Fig. 6). Such losses are described for phosphatidylcholine (Metlin spectra for MID182) but no spectra was available for LysoPC.

Finally, 4-pyridoxic acid was detected as [M + H] (*m*/*z* 184.06), along with a small peak corresponding to water loss (*m*/*z* 166.06), in both the standard solution and experimental sample at 60–61 s. Fragmentation spectra were superimposable and showed three major fragments at *m*/*z* 166.05, 148.04 and 138.05, as recorded in the Human Metabolome Database (HMDB00017) (Supplemental Fig. 7).

## Discussion

Although some molecular studies report biological effects after exposure to low doses of chemical compounds dispersed in the environment (Xu et al. 2013; Bonvallot et al. 2013; Rochester 2013; Dudka et al. 2014), few have described the biological mechanisms underlying the effects of radionuclides. The metabolomic approach appears relevant for improving knowledge in this field: it is very sensitive and holistic, because it measures the end products of the metabolism, close to the individual phenotype. With regards to the peculiar issue of radionuclides intake, additional studies performed with cesium 137 or strontium 90 have also described specific metabolic profiles that demonstrated the interest of untargeted metabolomics in the field of radioprotection (Goudarzi et al. 2016; Grison et al. 2012, 2013). Furthermore, studies performed in the field of external ionizing radiations found similar interests (Goudarzi et al. 2014; Johnson et al. 2012). Targeted studies focused on several biological parameters, but not on the whole metabolome, also reported molecular imbalances consistent with the present metabolomics results (Dublineau et al. 2014).

The overall aim of this study was to discover markers of low-dose exposure to uranium. Our initial goal, therefore, was to confirm our previous results, namely the biological response of rats to chronic low-dose contamination (40 mg L^−1^) for 9 months (Grison et al. 2013). As previously shown in the first cohort, our metabolomic approach enabled robust discrimination of exposed from control animals, even though standard clinical analyses revealed no significant differences between them. This confirms the analytical robustness of metabolomics in the field of low doses exposure.

The second goal of this study was to assess the minimal dose required to detect a measurable biological effect of uranium contamination in urine. We observed a shift in the metabolic profiles of the rats at a dose as low as 0.15 mg L^−1^ (“C dose”). It should be noted that this dose is only 5 times higher to the maximum dose recommended by the 2013 WHO drinking-water guideline (0.03 mg L^−1^) and that it is able to induce a measurable metabolic disruption after 9 months of contamination, and after only 6 months for the highest contamination dose tested (40 mg L^−1^). This could suggest that the threshold for a dose effect in urine in our experimental model would lie between 0.015 and 0.15 mg L^−1^. This analytical approach thus appears to be quite suitable for studies focused on low and very low doses of environmental pollutants such as radionuclides. Indeed, this work showed that metabolomics is able to identify contaminated rats despite normal level of clinical chemical markers. At the “D-dose” (1.5 mg L^−1^), our approach no longer discriminated between the control and contaminated groups at 6 or 9 months, although it had done so after 3 months of exposure. This nonlinear effect observed could constitute the threshold of response above which physiological tolerance is impaired, and below which biological response is related only to non-detrimental exposure. Such a dose dependent cellular adaptive mechanism has been described for both low doses (Calabrese 2004) and low-dose irradiation (Tang and Loke 2015). However, this issue must be deepened in future work to allow conclusions.

The third objective of our study was to identify a panel of urinary features highly associated with chronic exposure to low doses of natural uranium with the ultimate aim of discovering potential biomarkers of exposure. According to the results of the present study and to those of a recent study (Bonvallot et al. 2013), metabolomics confirms its relevance in the field of low dose environmental exposures to find new biological markers of low dose chronic exposure. By using the top-ranked features shared in the different statistical models discriminating control and contaminated rats (for all contamination doses and durations), we were able to select 11 features independently of doses (Fig. 3). To evaluate the possibility of using them as biomarkers of uranium exposure, we assessed their robustness by calculating their ROC curves and AUC values. The latter ranged from 58 to 80 %, which is good but not good enough to assure the use of these features alone as a diagnostic biomarker. On the other hand, the composite score built on these 11 features had AUC values ranging from 83 to 96 % (depending on the dose), which is more than satisfactory and suggests the value of using a combination of a few biomarkers instead of just one to develop a diagnostic test of exposure. As an example, this methodological approach was recently applied successfully to insulin resistance and prediabetes (Cobb et al. 2013). Such a diagnostic test for uranium exposure would be valuable, not only for screening exposed populations, but also for monitoring immediate and persistent metabolic changes among them, as a tool for early diagnosis of any disorders or even any risk of pathology induced by this chronic low-dose exposure.

Of course, the use of these features is not sufficient to build a diagnostic test because this requires identifying and quantifying metabolites. Accordingly, we confidently identified 5 metabolites (scores of 11), including N1-methylnicotinamide (NMN), N1-methyl-2-pyridone-5-carboxamide (NMPC), and 4-hydroxyphenylacetylglycine. The latter is involved in tyrosine metabolism and its concentration increases in the urine of contaminated rats (1.5 times on average). NMN and NMPC are involved in the metabolism of nicotinate and nicotinamide; urinary concentration of NMPC (the oxidation product of NMN) increases with contamination (1.5 times on average) while NMN concentration decreases (4.2 times on average). Our previous cohort study had already identified NMN (Grison et al. 2013), the concentration of which varied in the same direction. NMN clearance by kidneys is known to decrease in experimental renal failure (affecting the renal tubule) induced by chemical substances such as uranium nitrate, injected in rats at an acute high dose of 5 mg kg^−1^ (Shim et al. 1984). Another study reported a cortical uptake of NMN in rats after a single uranium nitrate injection at doses of 6, 1 and 0.5 mg kg^−1^ (Hirsch 1972). Recently, NMN was proposed as an endogenous probe for the evaluation of organic cation secretion in proximal tubules and of drug interactions with renal organic cation transporters (Ito et al. 2012; Müller et al. 2015). Moreover, uranium ingested chronically through drinking water at a dose of 40 mg L^−1^ accumulates primarily in the proximal tubules during the first 12 month and thereafter can be detected in all other segments of the nephron (Tessier et al. 2012). Finally, it has been reported that a single high dose of uranium (10 mg kg^−1^ in rats) causes structural damage to the renal proximal tubules (Haley et al. 1982). In this as in our previous study (Grison et al. 2013), NMN concentration decreased in the urine of the chronically contaminated rats, although these studies used lower doses of uranium than those cited above. In our experiments, the contaminated rats ingested on average only 1.7 mg day^−1^ kg^−1^ through drinking water (40 mg L^−1^) and no more than 0.4 % of it goes to the blood compartment. As with previous acute higher doses, chronic low-dose contamination by uranium also appears to decrease NMN renal clearance. One hypothesis to explain this reduction in the urinary concentration of NMN might be that uranium interacts with the renal secretion mechanisms of organic cation (Supplemental Fig. 8): uranium intake at a very low dose appears able to modify organic cation clearance without any renal tubular toxicity. This result might be interpreted as an early functional disorder in the kidneys that might lead to later morbidity. It also raises the question of the risk of drug-uranium interactions, which might lead to the onset of nephropathies. As a significant example of a possible functional effect in kidney, another study based on the effects of acetaminophen administration to rats chronically exposed to depleted uranium (Gueguen et al. 2007) has showed an increase of this last in plasma of rats exposed with uranium. According to our present hypothesis, this observation could be a consequence of drug-uranium interaction in kidneys, therefore changing the pharmacokinetics of acetaminophen. In any case, this observation seems to support the utility of these metabolites as sentinels for detecting early kidney disorders induced by uranium contamination, before any clinical signs appear. This study also demonstrates the interest of an omics approach to discover markers associated with biological low dose effects, such as the discovery of NMN as a candidate marker of renal function in the case of uranium intake. In conclusion, metabolomics studies focusing on the effects of either ionizing radiation or radionuclides intake has revealed, specific signatures of such exposures (Goudarzi et al. 2016; Grison et al. 2013; Johnson et al. 2012; Grison et al. 2012; Lestaevel et al. 2016). The present study adds further evidence that untargeted metabolomics could be a powerful approach to investigating low-dose ionizing and chemical effects in the field of radiotoxicology and might be successfully extended to molecular epidemiological studies assessing radiological hazards. This work must be completed with additional experimental studies (i) to test additional biological matrices, (ii) to validate the nonlinear dose–effect response observed in urine and (iii) to examine major confounding factors such as species, gender and age (Clayton and Collins 2014; Slupsky et al. 2007). Our experimental study suggests the possibility of developing a diagnostic test sufficiently sensitive to screen and monitor populations exposed chronically to very low doses of radionuclides likely to cause subtle biological effects. It would also help to answer societal questions about environmental exposures and low-dose risks. The results displayed also allowed to provide some explanations with regards to the biological mechanisms triggered by low dose uranium exposure and even the risk of adverse effects in organ function. Finally, it could also help to provide epidemiological data to improve public health regulation and thereby contribute to updating future health standards.

## Electronic supplementary material

Below is the link to the electronic supplementary material.

Supplementary material 1 (PPTX 345 kb)

Supplementary material 2 (PPTX 94 kb)

Supplementary material 3 (PPTX 70 kb)

Supplementary material 4 (PPTX 70 kb)

Supplementary material 5 (PPTX 71 kb)

Supplementary material 6 (PPTX 77 kb)

Supplementary material 7 (PPTX 76 kb)

Supplementary material 8 (PPTX 139 kb)

Supplementary material 9 (PPTX 64 kb)
