# Supplementary material for: Metabolomics reveals dose effects of low-dose chronic exposure to uranium in rats: identification of candidate biomarkers in urine samples
Source: Metabolomics. 2016 Sep 15;12(10):154. doi: 10.1007/s11306-016-1092-8 (PMC5025510; doi:10.1007/s11306-016-1092-8)
Supplement: Supplementary file 1 — Supplementary material 1 (PPTX 345 kb) [file 11306_2016_1092_MOESM1_ESM.pptx]

## Slide 1
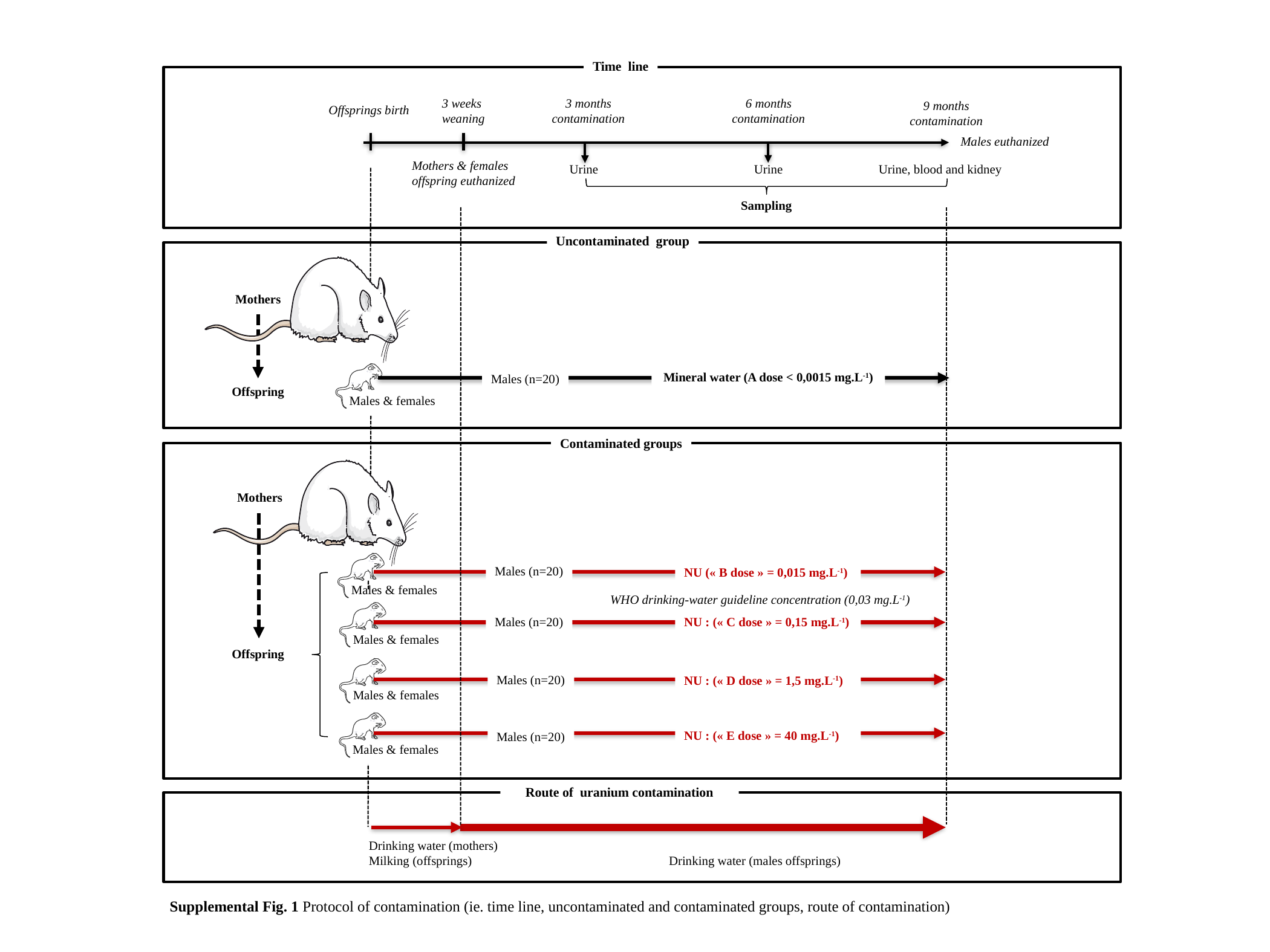

Time line
3 weeks
weaning
3 months
contamination
6 months
contamination
9 months
contamination
Offsprings birth
Males euthanized
Mothers & females
offspring euthanized
Urine
Urine
Urine, blood and kidney
Sampling
Uncontaminated group
Mothers
Males & females
Mineral water (A dose < 0,0015 mg.L-1)
Males (n=20)
Offspring
Contaminated groups
Mothers
Males & females
Males (n=20)
NU (« B dose » = 0,015 mg.L-1)
WHO drinking-water guideline concentration (0,03 mg.L-1)
Males & females
Males (n=20)
NU : (« C dose » = 0,15 mg.L-1)
Offspring
Males & females
Males (n=20)
NU : (« D dose » = 1,5 mg.L-1)
Males & females
NU : (« E dose » = 40 mg.L-1)
Males (n=20)
Route of uranium contamination
Drinking water (mothers)
Milking (offsprings)
Drinking water (males offsprings)
Supplemental Fig. 1 Protocol of contamination (ie. time line, uncontaminated and contaminated groups, route of contamination)
