# Supplementary material for: Metabolomics reveals dose effects of low-dose chronic exposure to uranium in rats: identification of candidate biomarkers in urine samples
Source: Metabolomics. 2016 Sep 15;12(10):154. doi: 10.1007/s11306-016-1092-8 (PMC5025510; doi:10.1007/s11306-016-1092-8)
Supplement: Supplementary file 2 — Supplementary material 2 (PPTX 94 kb) [file 11306_2016_1092_MOESM2_ESM.pptx]

## Slide 1
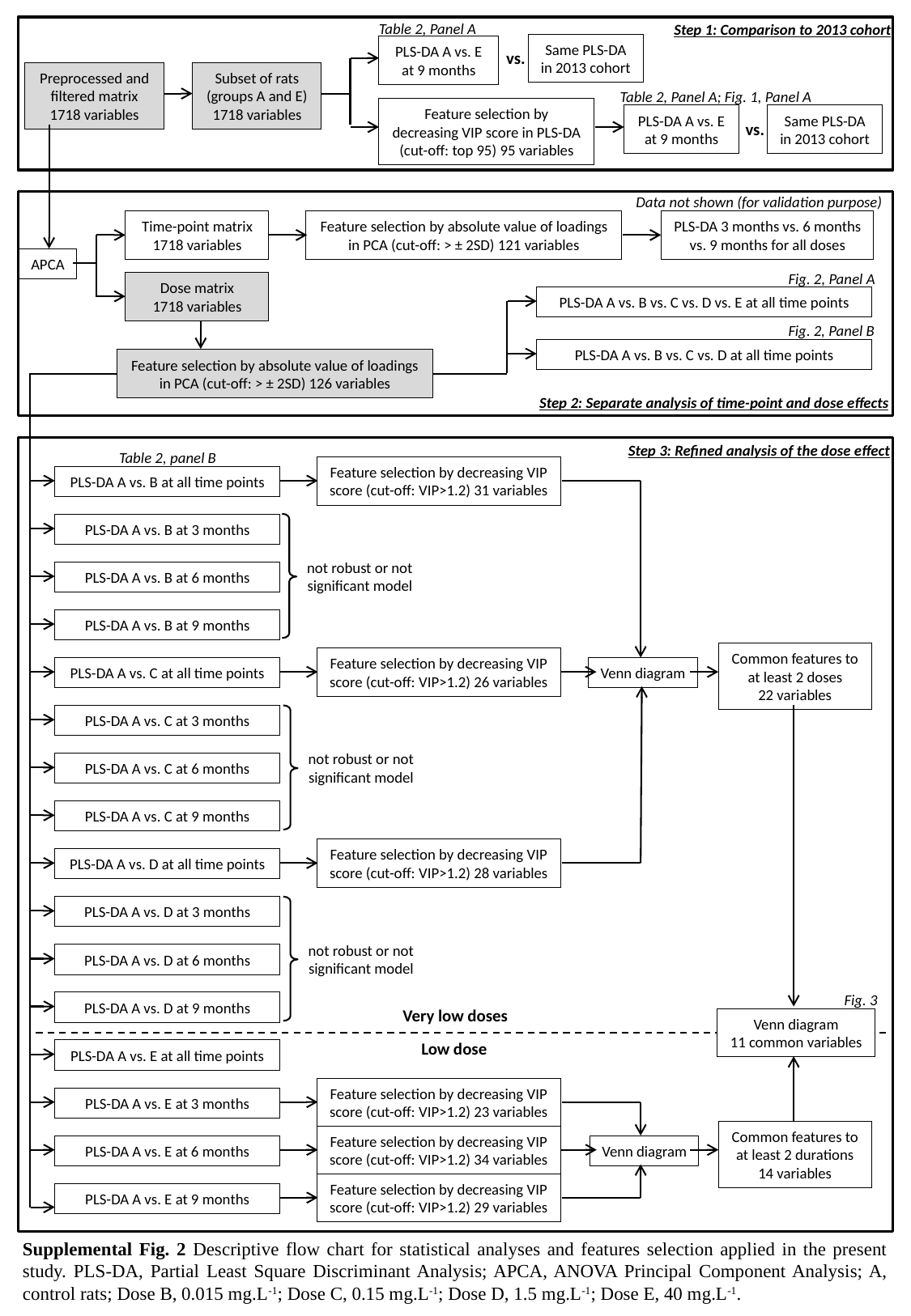

Table 2, Panel A
Step 1: Comparison to 2013 cohort
Same PLS-DA in 2013 cohort
PLS-DA A vs. E
at 9 months
vs.
Preprocessed and filtered matrix
1718 variables
Subset of rats (groups A and E) 1718 variables
Table 2, Panel A; Fig. 1, Panel A
Feature selection by decreasing VIP score in PLS-DA (cut-off: top 95) 95 variables
PLS-DA A vs. E at 9 months
Same PLS-DA in 2013 cohort
vs.
Data not shown (for validation purpose)
Time-point matrix
1718 variables
Feature selection by absolute value of loadings in PCA (cut-off: > ± 2SD) 121 variables
PLS-DA 3 months vs. 6 months vs. 9 months for all doses
APCA
Fig. 2, Panel A
Dose matrix
1718 variables
PLS-DA A vs. B vs. C vs. D vs. E at all time points
Fig. 2, Panel B
PLS-DA A vs. B vs. C vs. D at all time points
Feature selection by absolute value of loadings in PCA (cut-off: > ± 2SD) 126 variables
Step 2: Separate analysis of time-point and dose effects
Step 3: Refined analysis of the dose effect
Table 2, panel B
Feature selection by decreasing VIP score (cut-off: VIP>1.2) 31 variables
PLS-DA A vs. B at all time points
PLS-DA A vs. B at 3 months
not robust or not significant model
PLS-DA A vs. B at 6 months
PLS-DA A vs. B at 9 months
Common features to at least 2 doses
22 variables
Feature selection by decreasing VIP score (cut-off: VIP>1.2) 26 variables
PLS-DA A vs. C at all time points
Venn diagram
PLS-DA A vs. C at 3 months
not robust or not significant model
PLS-DA A vs. C at 6 months
PLS-DA A vs. C at 9 months
Feature selection by decreasing VIP score (cut-off: VIP>1.2) 28 variables
PLS-DA A vs. D at all time points
PLS-DA A vs. D at 3 months
not robust or not significant model
PLS-DA A vs. D at 6 months
Fig. 3
PLS-DA A vs. D at 9 months
Very low doses
Venn diagram
11 common variables
Low dose
PLS-DA A vs. E at all time points
Feature selection by decreasing VIP score (cut-off: VIP>1.2) 23 variables
PLS-DA A vs. E at 3 months
Common features to at least 2 durations
14 variables
Feature selection by decreasing VIP score (cut-off: VIP>1.2) 34 variables
PLS-DA A vs. E at 6 months
Venn diagram
Feature selection by decreasing VIP score (cut-off: VIP>1.2) 29 variables
PLS-DA A vs. E at 9 months
Supplemental Fig. 2 Descriptive flow chart for statistical analyses and features selection applied in the present study. PLS-DA, Partial Least Square Discriminant Analysis; APCA, ANOVA Principal Component Analysis; A, control rats; Dose B, 0.015 mg.L-1; Dose C, 0.15 mg.L-1; Dose D, 1.5 mg.L-1; Dose E, 40 mg.L-1.
