# Supplementary material for: Metabolomics reveals dose effects of low-dose chronic exposure to uranium in rats: identification of candidate biomarkers in urine samples
Source: Metabolomics. 2016 Sep 15;12(10):154. doi: 10.1007/s11306-016-1092-8 (PMC5025510; doi:10.1007/s11306-016-1092-8)
Supplement: Supplementary file 4 — Supplementary material 4 (PPTX 70 kb) [file 11306_2016_1092_MOESM4_ESM.pptx]

## Slide 1
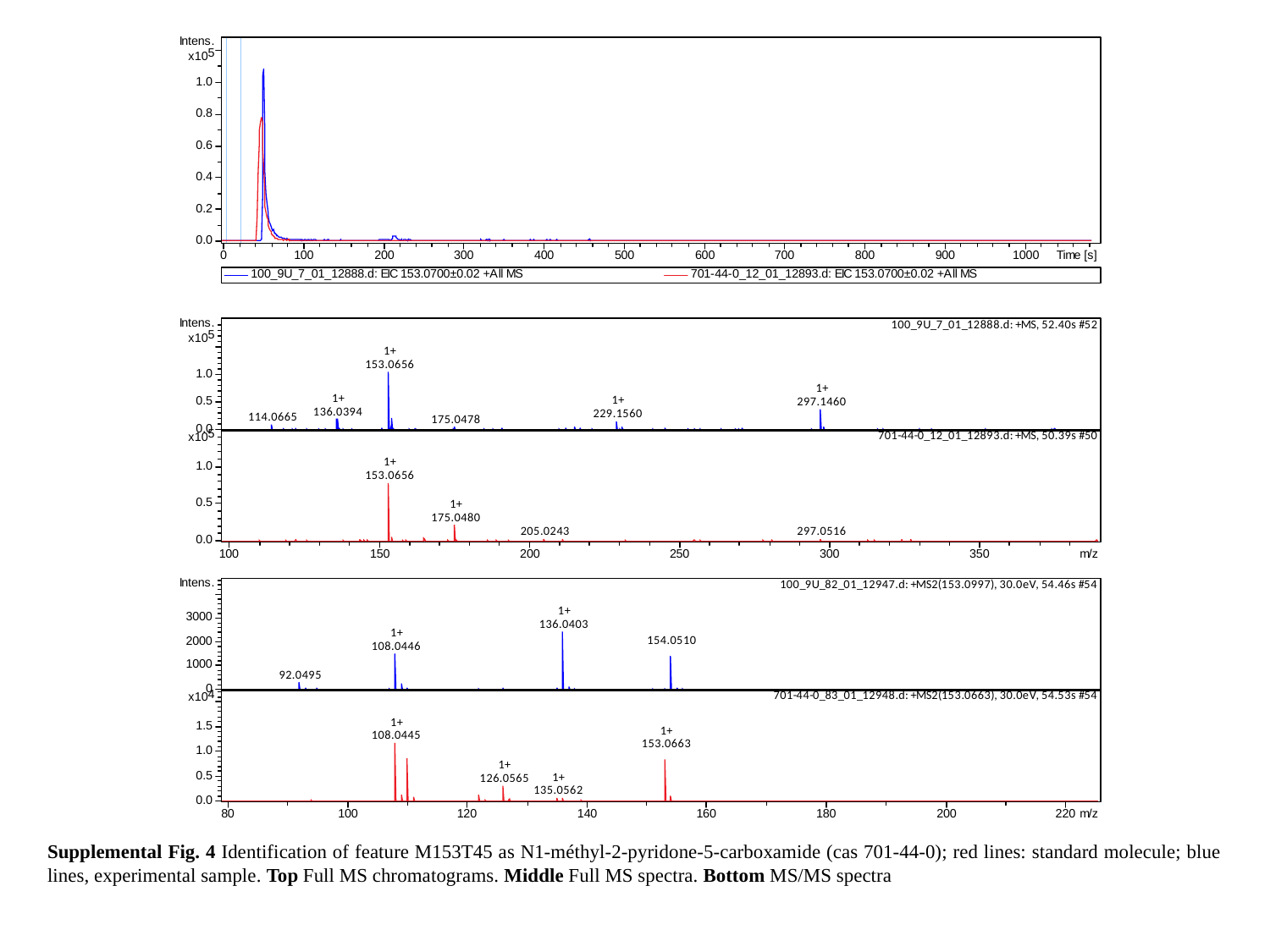

Supplemental Fig. 4 Identification of feature M153T45 as N1-méthyl-2-pyridone-5-carboxamide (cas 701-44-0); red lines: standard molecule; blue lines, experimental sample. Top Full MS chromatograms. Middle Full MS spectra. Bottom MS/MS spectra
