# Supplementary material for: Metabolomics reveals dose effects of low-dose chronic exposure to uranium in rats: identification of candidate biomarkers in urine samples
Source: Metabolomics. 2016 Sep 15;12(10):154. doi: 10.1007/s11306-016-1092-8 (PMC5025510; doi:10.1007/s11306-016-1092-8)
Supplement: Supplementary file 7 — Supplementary material 7 (PPTX 76 kb) [file 11306_2016_1092_MOESM7_ESM.pptx]

## Slide 1
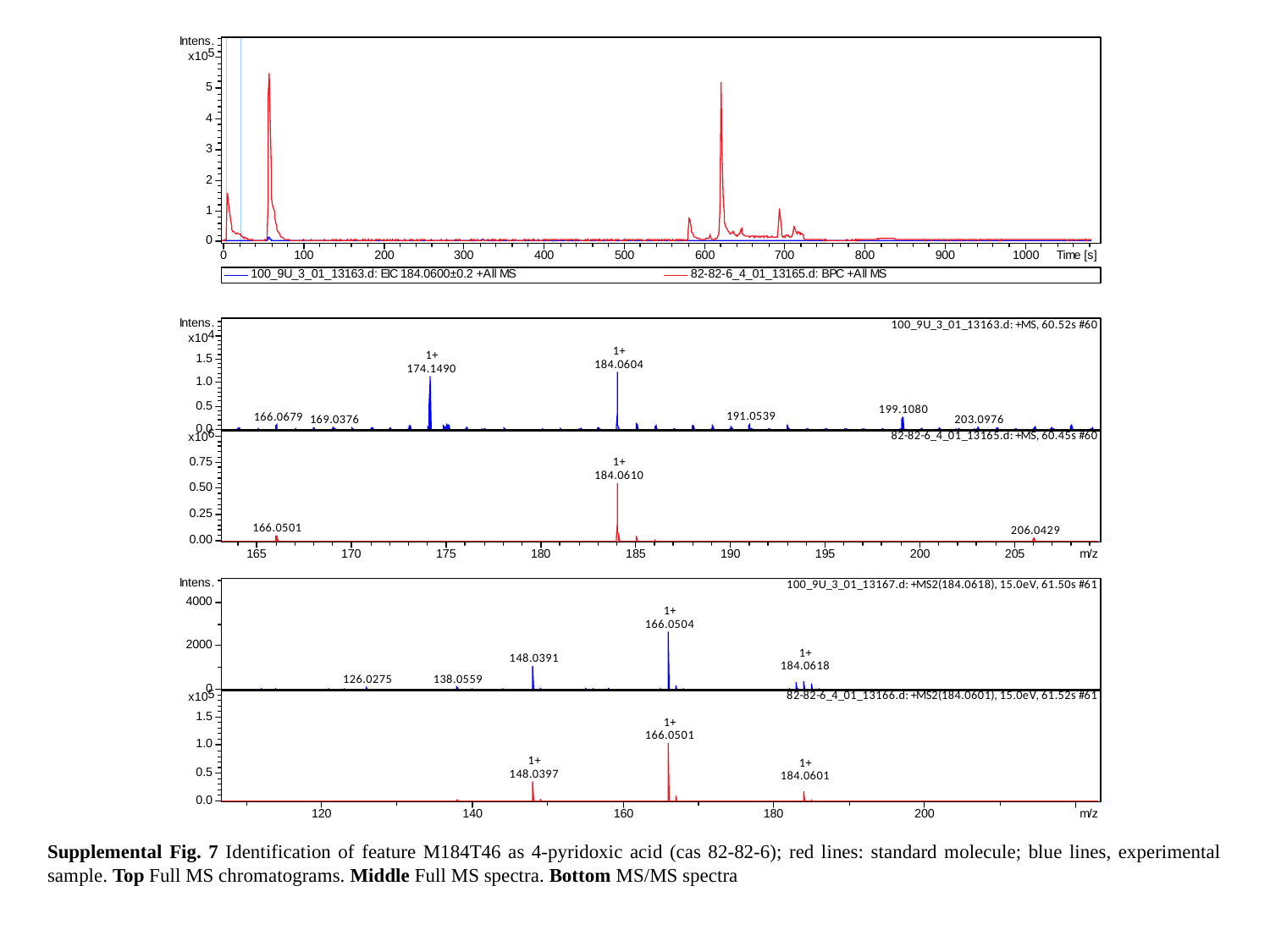

Supplemental Fig. 7 Identification of feature M184T46 as 4-pyridoxic acid (cas 82-82-6); red lines: standard molecule; blue lines, experimental sample. Top Full MS chromatograms. Middle Full MS spectra. Bottom MS/MS spectra
