# Supplementary material for: Metabolomics reveals dose effects of low-dose chronic exposure to uranium in rats: identification of candidate biomarkers in urine samples
Source: Metabolomics. 2016 Sep 15;12(10):154. doi: 10.1007/s11306-016-1092-8 (PMC5025510; doi:10.1007/s11306-016-1092-8)
Supplement: Supplementary file 8 — Supplementary material 8 (PPTX 139 kb) [file 11306_2016_1092_MOESM8_ESM.pptx]

## Slide 1
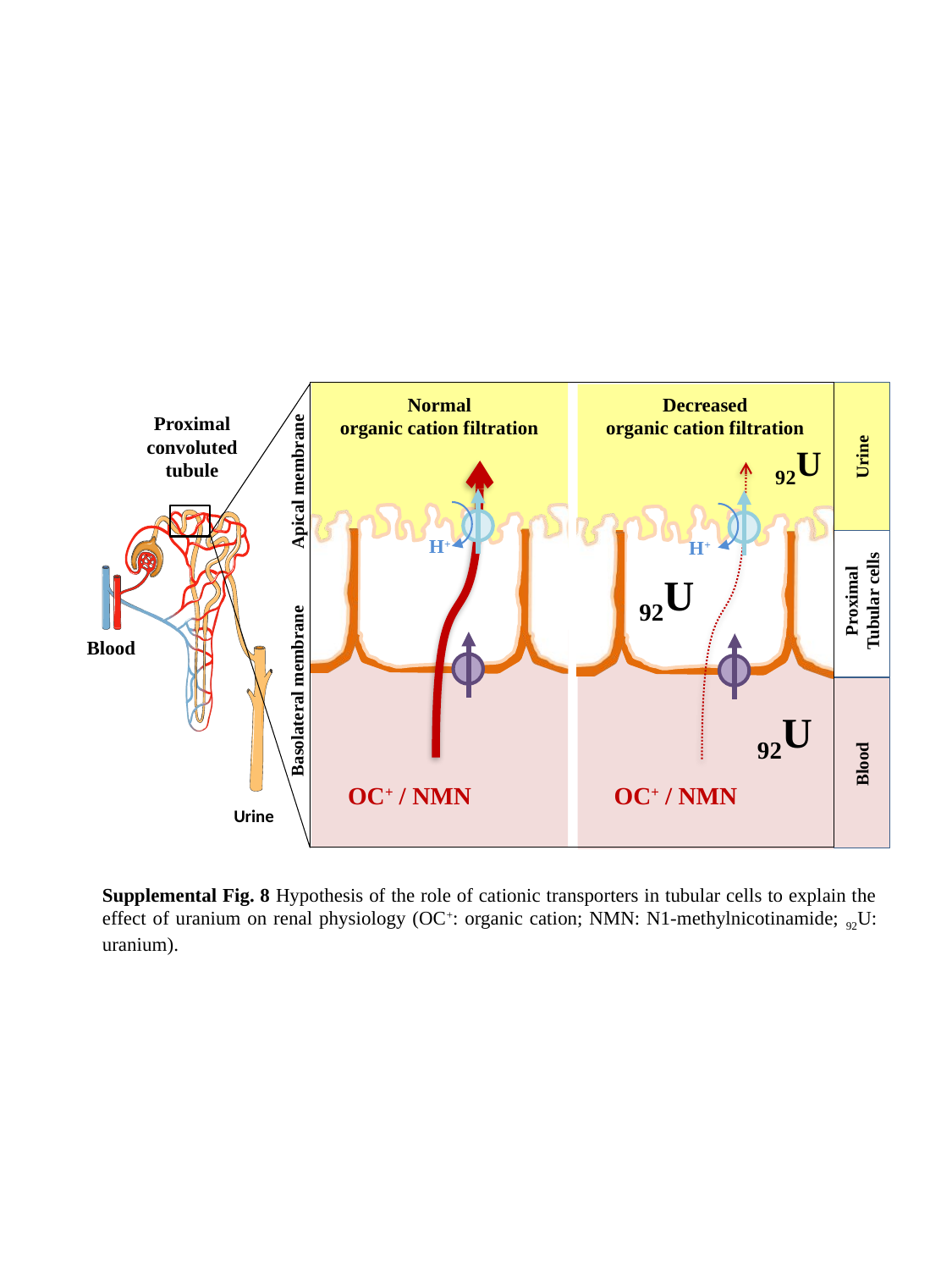

Normal
organic cation filtration
Decreased
organic cation filtration
Proximal
convoluted tubule
92U
Urine
Apical membrane
H+
H+
92U
Proximal Tubular cells
Blood
Basolateral membrane
92U
Blood
OC+ / NMN
OC+ / NMN
Urine
Supplemental Fig. 8 Hypothesis of the role of cationic transporters in tubular cells to explain the effect of uranium on renal physiology (OC+: organic cation; NMN: N1-methylnicotinamide; 92U: uranium).
