# Supplementary material for: Metabolomics reveals dose effects of low-dose chronic exposure to uranium in rats: identification of candidate biomarkers in urine samples
Source: Metabolomics. 2016 Sep 15;12(10):154. doi: 10.1007/s11306-016-1092-8 (PMC5025510; doi:10.1007/s11306-016-1092-8)
Supplement: Supplementary file 9 — Supplementary material 9 (PPTX 64 kb) [file 11306_2016_1092_MOESM9_ESM.pptx]

## Slide 1
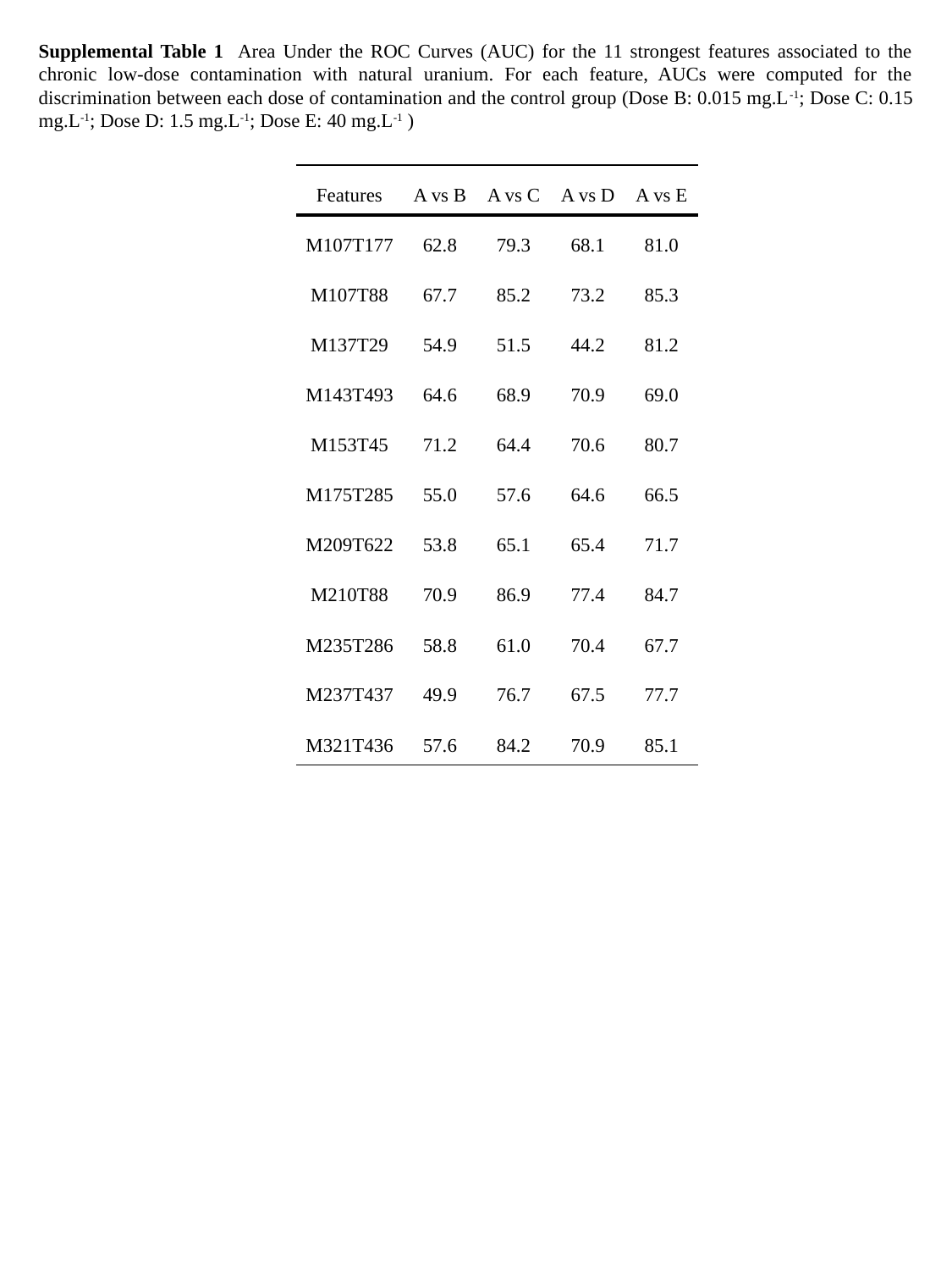

Supplemental Table 1 Area Under the ROC Curves (AUC) for the 11 strongest features associated to the chronic low-dose contamination with natural uranium. For each feature, AUCs were computed for the discrimination between each dose of contamination and the control group (Dose B: 0.015 mg.L-1; Dose C: 0.15 mg.L-1; Dose D: 1.5 mg.L-1; Dose E: 40 mg.L-1 )
| Features | A vs B | A vs C | A vs D | A vs E |
| --- | --- | --- | --- | --- |
| M107T177 | 62.8 | 79.3 | 68.1 | 81.0 |
| M107T88 | 67.7 | 85.2 | 73.2 | 85.3 |
| M137T29 | 54.9 | 51.5 | 44.2 | 81.2 |
| M143T493 | 64.6 | 68.9 | 70.9 | 69.0 |
| M153T45 | 71.2 | 64.4 | 70.6 | 80.7 |
| M175T285 | 55.0 | 57.6 | 64.6 | 66.5 |
| M209T622 | 53.8 | 65.1 | 65.4 | 71.7 |
| M210T88 | 70.9 | 86.9 | 77.4 | 84.7 |
| M235T286 | 58.8 | 61.0 | 70.4 | 67.7 |
| M237T437 | 49.9 | 76.7 | 67.5 | 77.7 |
| M321T436 | 57.6 | 84.2 | 70.9 | 85.1 |
